# High Injury Incidence Among Youth in the World's Largest Football Tournament

**DOI:** 10.1111/sms.70072

**Published:** 2025-05-16

**Authors:** Ida Lindman, William Högne, Gabriel Johansson, Josefin Abrahamson

**Affiliations:** ^1^ Public Health and Community Medicine, Institute of Medicine, Sahlgrenska Academy University of Gothenburg Gothenburg Sweden; ^2^ Region Västra Götaland, Research, Education, Development & Innovation Primary Health Care Gothenburg Sweden; ^3^ Department of Health and Rehabilitation, Institute of Neuroscience and Physiology at Sahlgrenska Academy, University of Gothenburg Gothenburg Sweden; ^4^ Orthopaedic Research Unit Sahlgrenska University Hospital Gothenburg Sweden

**Keywords:** football injuries, injury incidence, sports injuries, youth football

## Abstract

Gothia Cup is the world's largest youth football (soccer) tournament, held annually since 1975. In 2024, 33 060 players from over 72 countries participated in 4820 matches. Although studies have examined injuries among youth football, research during tournaments remains limited. This prospective study aimed to assess the incidence of injuries among youth football players in Gothia Cup. Secondary objectives included comparing injury by age, sex, and across days of the tournament. All visits at medical tents were recorded. An injury was defined according to FIFA's suggestion as any physical condition that prompted a football player to seek medical attention during the tournament. A total of 1184 injuries were reported, resulting in an overall injury rate of 15.4 injuries/1000 player hours (95% CI, 14.5–16.2). Girls experienced a higher injury rate than boys (16.7, 95% CI 15.0–18.3 vs. 14.7, 95% CI 13.7–15.7 injuries/1000 player hours). Injury incidence increased progressively throughout the tournament, with the lowest rate on the first day (8.8 injuries/1000 player hours, 95% CI 7.3–10.3) and highest on the last day (37.6 injuries/1000 player hours, 95% CI 25.5–49.7). Incidence varied significantly across age groups, with the youngest players (aged 11 and 12 years) exhibiting the highest injury rates (27.7, 95% CI 21.6–33.8 and 25.7, 95% CI 21.0–30.5, injuries/1000 player hours, respectively). Lower extremity injuries were most common. However, head injuries accounted for 7% of all injuries. These findings highlight the injury patterns in a large‐scale, six‐day youth football tournament, providing valuable insights for the development of medical strategies in future football tournaments.

## 1 Introduction

Football (soccer) is considered the most popular sport in the world, with over millions of individuals playing globally [1, 2, 3]. According to FIFA's report, more than half of the registered players are under the age of 18 years [1]. Participating in football is further driven by the numerous health benefits such as increased health including improved cardiovascular health, body composition, and muscular strength [4, 5].

Although youth football is often seen as a beneficial, players in this age group remain vulnerable to injuries [3, 6, 7]. In fact, youth football players have a high probability of sustaining injuries [8]. Football is a complex contact sport that requires high‐intensive movements such as rapid acceleration and deceleration, jumping and landing, and frequent changes in direction, potentially increasing the risk of injury [8, 9, 10, 11]. The most affected injury location is the lower limb, including the knee and ankle [12, 13]. There are several studies regarding injuries among youth football players with an incidence between 0.61 and 39.2 injuries per 1000 h of football [10, 14, 15, 16, 17, 18]. The various incidences depend both on the definition of injury and whether the study was seasonal or during games. A higher incidence has further been shown during games (2.2–80.0 injuries/1000 match hours) than training (0.69–11.9 injuries/1000 training hours) [8, 9, 10]. However, most of these studies have been conducted over seasons and in elite youth players, and only a sparse number of studies have been on tournaments [19, 20, 21]. Furthermore, the studies considering tournaments were conducted over 10 years ago, indicating the need for updated studies. While there have been considerable strides in the realm of football injury prevention, such as the 11 + injury prevention programme [22, 23], further prevention strategies need to be taken during tournaments. Hence, understanding injury incidence during larger tournaments is crucial for designing targeted prevention programs and ensuring that adequate medical support is available at large‐scale tournaments.

Annually since 1975, Gothenburg, Sweden has hosted the world's largest youth football tournament, Gothia cup, with 4820 games spanning over six consecutive days. In 2024, the tournament included 1911 teams from 72 nations. Using patient charts from this tournament, this study aimed to investigate incidence injuries among youth football players attending Gothia cup 2024. The secondary aims were to compare injury incidence between age, sex, and across the duration of the tournament.

## 2 Methods

### 2.1 Study Design

This study was a prospective observational study.

### 2.2 Ethics

This study was approved by the Swedish Ethical Review authority with diary number 2023–06647‐01. All data were collected anonymously with no personal identification recorded, ensuring the confidentiality and privacy. Athletes and guardians, when applicable, were informed about the anonymously collected data. The study adhered to ethical guidelines of the Declaration of Helsinki.

### 2.3 Study Participants

All football players aged 11–19 years old participating at Gothia cup during the tournament in 2024 were eligible for the study. During the tournament, several on‐site medical tents were set up. These tents were easily accessible and were free‐of‐charge to all participants. The staff in these tents consisted of trained first‐aid personnel (nurses, nurse assistants, etc.) and physiotherapists. All players who sought medical attention from any of these healthcare personnel were registered. However, to assess injury incidence and prevalence, all registered players in the tournament were included in these analyses.

### 2.4 Data Collection

All visits at the medical tents were recorded in a custom‐designed application specifically developed for this tournament. For all contacts, a patient chart was completed in the application comprising the players age, sex, nationality, injury location, time for treatment, and action taken (e.g., the injured player left after treatment, medical transport picked up the injured, ambulance arrived etc.). Contacts related to an injury or illness not directly associated with playing football were also documented and treated; however, they were excluded from the analysis in this study. This approach of data collection has previously been used to evaluate injuries in similar tournaments [19, 20]. Injury prevalence was expressed as the number of injured players during the tournament divided by the number of players at risk during the tournament, and injury incidence as the number of injuries divided by 1000 player hours.

#### 2.4.1 Definition of Injury

An injury was defined as any physical condition that prompted a football player to seek medical attention during the tournament. This definition has been commonly used and recommended in a consensus statement established by FIFA Medical assessment and Research Centre [24]. By the use of this definition, it offers future tournament organizers essential guidance for planning medical support appropriately.

### 2.5 The Tournament

Gothia cup is played according to the rules of FIFA [25]. The games were played on grass or artificial turf on 142 different arenas at different venues. All venues had medical staff/medical tents. The teams were divided into age groups, with males and females playing separately in all age groups. The tournament was structured in two rounds. The first round consisted of a group play where all teams within a group met each other in a series, and the second round consisted of a playoff with knock‐out competition. The two best teams in each group reach playoff A and the other teams reach playoff B. However, the youngest (11 years old) have no playoff. The number of players differed between age groups. Players aged 11–12 years played 7‐a‐side, players aged 13 played 9‐a‐side, and all other age groups played 11‐a‐side. Furthermore, playing times differed depending on age and whether it was group play or playoff. The youngest (11–14 years) played 2 × 20 min, prolonged to 2 × 25 during playoffs, whereas the older (15–19 years) played 2 × 25 min, prolonged to 2 × 30 during playoffs.

### 2.6 Statistics

The data were analyzed using IBM SPSS Statistics for Mac, version 29.0 (IBM Corp., Armonk, NY) and Microsoft Excel. For injury prevalence and incidence, the corresponding 95% confidence interval (CI) was calculated according to Knowles et al. [26]. Player hours were calculated as the number of matches in each age group multiplied by the corresponding match time multiplied by the number of players on the field in each match. The independent *t*‐test was used for comparisons of continuous variables and the Chi^2^ test for comparisons between categorical variables. To compare injury incidence between groups (girls vs. boys, age groups, and day of the tournament), injury incidence rate ratios (IRRs) were calculated. The IRRs were calculated, according to Knowles et al. [26], as the ratio of two incidence rates (injuries/1000 player hours) (See Supporting Information for formulas). Regarding the comparison of age, age groups were categorized according to the divisions used in the tournament. Statistical significance was considered present if the 95% CI between two groups did not overlap or had a *p*‐value < 0.05. For the IRRs, the difference in injury incidence was considered statistically significant if the 95% CI did not include 1.00.

## 3 Results

A total of 4820 games were played by 33 060 players (31% girls) (Table 1). The total number of reported injuries during the tournament was 1184, of which 384 (32%) were among girls, 794 (67%) among boys, and six (1%) with unreported sex. Mean age at the time of reporting an injury was 14.6 (SD 1.9) years, with no difference between the sexes. Most injured players were from Sweden (35%), while the remaining Nordic countries accounted for 7% and the rest of Europe for 26%. Sixteen percent were from North America, 8% from South America, and 4% from Africa and Asia, respectively.

**TABLE 1:** Demographics and injury data in total and stratified by sex.

	Total	Girls	Boys
Games, *n*	4820^a^	1385	3379
Total number of players, *n* (%)	33 060	10 249 (31.0%)	22 811 (69.0%)
Total playing h, (mean hours/player)	77 021 (2.3)	22 938 (2.0)	53 690 (2.3)
Number of injuries, *n* (%)	1184 (100%)^b^	384 (32.6%)	794 (67.4%)
Mean age at injury, years (SD)	14.6 (1.9)	14.5 (2.0)	14.7 (1.8)
Injury prevalence, % (95% CI)	3.6 (3.4–3.8)	3.7 (3.4–4.1)	3.5 (3.2–3.7)
Injuries/1000 player hours, (95% CI)	15.4 (14.5–16.3)	16.7 (15.0–18.3)	14.7 (13.7–15.7)

Abbreviations: CI, confidence interval; h, hours; *n*, number; SD, Standard deviation.

^a^ Fifty six games were played by special teams with girls and boys mixed.

^b^ Six players had unreported sex.

Of the registered injuries, 1% (*n* = 9) were so severe that an ambulance was required, and in 7% (*n* = 82) of the cases, the players were transported independently or with the help of medical transport to a physician for further evaluation. Head injuries accounted for 33.3% (*n* = 3) of the injuries where an ambulance was required and for 17.1% (*n* = 14) where the player was transported for further evaluation. However, upper extremity injuries (*n* = 25, 30.4%) and foot/ankle injuries (*n* = 22, 26.5%) were more common in athletes needing transportation for further evaluation. Five of the nine injuries requiring an ambulance occurred during day 2 of the tournament, while most injuries requiring other transportation for further evaluation occurred during day 3 (*n* = 22, 26.9%) and day 4 (*n* = 21, 25.7%).

Table 1 shows demographics and injury data. The injury prevalence for the tournament in total was 3.6% (95% CI 3.4–3.8%), with no significant difference between girls (3.7%, 95% CI 3.4–4.1%) and boys (3.5%, 95% CI 3.2–3.7%) (*p* = 0.2). There were in total 15.4 (95% CI 14.5–16.3) injuries/1000 player hours, with more injuries reported for girls compared with boys (16.7, 95% CI 15.0–18.3 vs. 14.7, 95% CI 13.7–15.7) (Table 1). This was though not statistically significant, and the IRRs for boys vs. girls were 0.88 (95% CI 0.78–1.00). The total number of playing hours was a total of 77 021 playing hours (2.3 h/players). The number of playing hours for girls and boys can be seen in Table 1.

**FIGURE 1:**
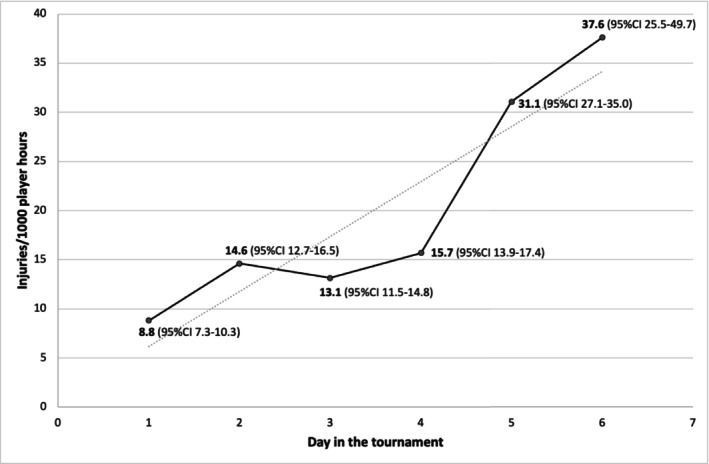
Injury incidence defined as injuries/1000 players hours between the different days in the tournament. The incidence differed significantly between each day of the tournament. Plotted line = linear trend line.

The injury incidence differed significantly between almost all days of the tournament, with the highest IRR between day 6 and day 1 (4.3, 95% CI 3.0–6.1). The injury incidence increased as the tournament went on, with the lowest injury incidence on day 1 (8.8 injuries/1000 player hours, 95% CI 7.3–10.3) and the highest on the last day, day 6 (37.6 injuries/1000 players hours, 95% CI 25.5–49.7) (Figure 1). See Supporting Information for additional IRRs with 95% CI between all days in the tournament.

**FIGURE 2:**
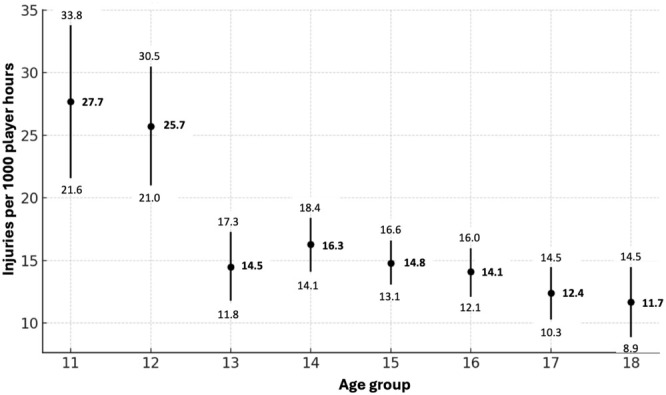
Injuries stratified by age groups.

Figure 2 Shows injuries/1000 player hours between the different age groups. The youngest age groups (11 and 12 years) had a significantly higher incidence of injuries (27.7, 95% CI 21.6–33.8, and 25.7, 21.0–30.5 injuries/1000 player hours respectively), compared with the other age groups (13–18 years) (11.7–16.3, 95% CI 8.9–18.4, injuries/1000 player hours). The IRRs between the youngest age groups (11 and 12 years) and the other age groups (13–18 years) were between 0.4–0.6 (95% CI 0.3–0.8). For the other age groups (13–18 years), the injury incidence was similar and not statistically significant. For additional IRRs with 95% CI between all age groups, see Supporting Information.

**FIGURE 3:**
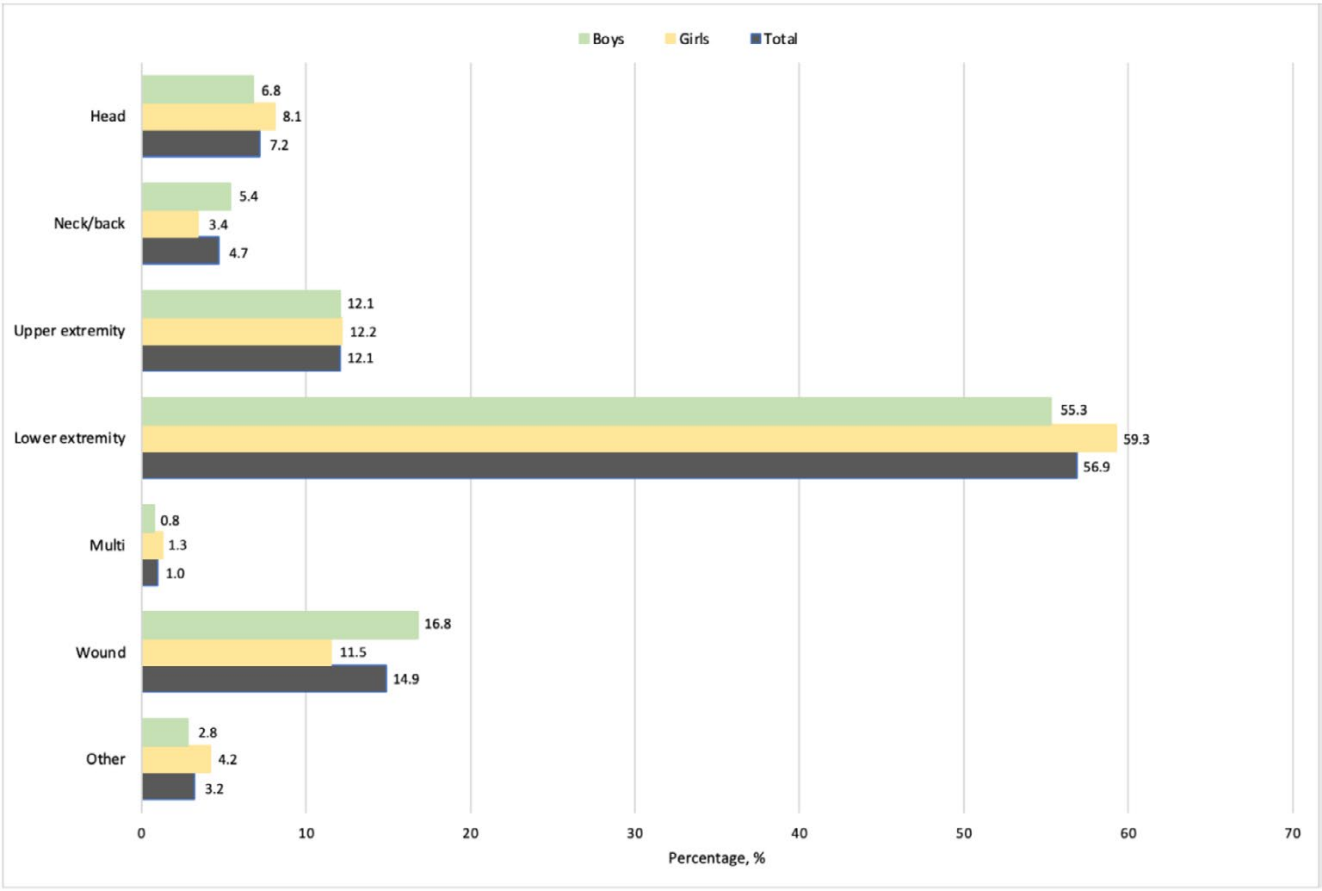
Injury location in total and stratified by sex.

The most common injury location was to the lower extremity, with no differences between the sex (Figure 3). The foot/ankle was most injured in girls (31%), while the knee and foot/ankle were almost similarly common in boys (27% and 25% respectively). Head injuries accounted for 7% of all registered injuries.

## 4 Discussion

This prospective study is one of the largest football studies including 33 060 players and 4820 games during one tournament over the course of six consecutive days. While there is a tremendous number of previous studies on football, this is one of the few studies considering youth football tournaments.

A total of 15.4 injuries/1000 player hours was found, which is comparable with previous studies considering youth football tournaments [19, 20]. Kolstrup et al. reported nearly identical injury incidence, 15.3 injuries/1000 player hours, as observed in the present study, during three tournaments each lasting 4 days in Denmark [20]. Additionally, another study examining injury data from a decade in an annual youth football tournament in the United States found injury incidence varied between 7.6 and 20.1 injuries/1000 player hours [19]. However, the latter study, which included data between 1988 to 1999, may be less comparable due to advancements in football, both in terms of training methods, equipment, and implementation of prevention programs.

When compared to previous studies on youth football players conducted outside of tournament settings, the incidence rate found in this study is higher [7, 27, 28]. These earlier studies typically report data from regular seasons and include training session data. Injury incidence has shown to be five to seven times higher during matches compared with training, with injury incidence during matches similar to that observed in this study [9, 15]. This highlights the importance of preventive strategies during matches and tournaments.

Higher injury rates were observed overall among girls compared with their male counterparts (16.7 compared with 14.7 injuries/1000 players hours). This finding aligns with previous studies, which have reported elevated injury incidence in girls [19, 20, 29]. Particularly, anterior cruciate ligament tear (ACL) is widely known to be more common among girls [30, 31]. Several factors may contribute to this disparity between sexes. Physiological aspects such as differences in muscle mass, joint laxity, and hormonal fluctuations may play a role in increasing the risk of injury in females [9, 32]. Additionally, biomechanical differences in movement patterns between sexes may contribute to the higher injury rates observed among girls. Girls often exhibit different landing mechanics and knee alignment, which could place more stress on certain joints, thereby increasing the likelihood of injury [30]. Furthermore, disparities in training, including techniques and strength conditioning between boys and girls, as well as differences in neuromuscular control, hormonal influences, and physical maturation, may influence injury prevalence [32]. Several programs have been implemented in order to reduce the injury burden among female football players, with various results [32]. However, as female football is the fastest growing sport in the world, further actions need to be taken.

Another interesting finding in the present study was the difference in injury rates over the course of the tournament, where it increased as the tournament went on, with the lowest injury incidence on day 1 (8.8 injuries/1000 player hours) and the highest on the last day, day 6 (37.6 injuries/1000 player hours). This pattern mirrors the results from Kolstrup et al. [20]. One potential explanation for this trend is the cumulative effect of fatigue as players engage in several games coupled with limited recovery time. The increase in injury rates observed in this study may, therefore, reflect the growing exhaustion and reduced ability to recover between games. Additionally, as the tournament advances, the intensity of competition typically escalates, which could further elevate injury risk. Another possible explanation can be that some injuries are initially neglected due to the belief that they will recover on their own or that the player may not risk losing their place in the starting team. This could result in some players seeking help later in the tournament. It is worth noting that this study did not distinguish the severity of injury. Nonetheless, the finding that injury rates escalated as the tournament advanced highlights the importance of incorporating adequate rest periods, recovery strategies, and injury prevention measures, especially in tournaments where players participate in multiple matches over a short period.

The lower limb including knee and ankle was the most commonly injured body part. This is expected and comparable to all previous studies [7, 8, 10, 12, 20, 33]. Notably is the high frequency of injuries toward the head, which accounted for 7% of all the injuries. Furthermore, of the registered head injuries, 3.9% required ambulance transport to the hospital and 12.9% sought physician or other healthcare professional for further evaluation, compared with 0.7% and 6.1% for all injuries. Depending on definition and included population, an incidence of 4%–22% of head–neck injuries has been reported [12, 16, 34]. While the youth brain can be particularly vulnerable to head injury, an incidence of 7% is of highest concern.

Younger players were more prone to injury, with 11‐year‐olds experiencing 27.7 injuries per 1000 player hours. This rate decreased with age, and 18‐year‐olds had the lowest incidence, with 11.7 injuries per 1000 player hours. While other studies have reported opposite results [10, 16], with higher injury among older players, the results of this study are not surprising. Younger players may be less accustomed to the intense demands of multiple games in a single day, coupled with insufficient rest and cumulative fatigue, making them more susceptible to injuries during tournaments. In contrast, previous studies conducted during the whole season and including training may reflect the seasonal overall risk for older players, who generally have higher training volumes and more games. Kolstrup et al. [20], the only similar study including a large‐scale tournament, found the same results as in this study.

### 4.1 Strengths and Limitations

This is one of the few studies regarding youth football tournament with one of the largest cohorts and participants from all over the world. However, it is not without limitations. Severity of injury was not taken into account. Several studies measure severity based on absence from play, which was not possible during this study. As suggested by previous consensus statement, and in line with the purpose of this study injury was defined as “seeking medical attention” [24]. However, it is possible that some players sustained injuries on the field and sought medical attention elsewhere. While this is a possibility, the medical tents were strategically placed throughout the tournament to ensure easy access to care. Therefore, players who may have sought medical assistance elsewhere without first visiting the medical tents are considered negligible in this analysis. The data in this study is based on player hours during the tournament. However, some of the reported injuries may have occurred during training or warm‐up sessions between games. While no specific data on these occurrences exist, it is believed that their impact on the overall conclusions is likely to be minimal. Furthermore, there is also potential over−/underestimation of exposure due to variations in actual playing time, such as substitutions and early match terminations, which were not accounted for in the standardized player‐hours calculation. Although, this issue is considered to be minor and unlikely to significantly affect the overall findings.

## 5 Conclusions

During a large‐scale, 6‐day youth football tournament, the overall injury rate was 15.37 injuries per 1000 player hours. The incidence was higher among girls than boys, and younger players had the highest injury rates, with the incidence decreasing as age increased. Additionally, the injury rate rose progressively throughout the tournament, with the highest incidence observed on the final day. These findings highlight the injury patterns and provide valuable insights for the development of medical strategies in future youth football tournaments.

## Ethics Statement

This study was approved by the Swedish Ethical Review authority with diary number 2023‐06647‐01.

## Conflicts of Interest

One of the authors (I.L.) has served as a medical doctor during the tournament in question; however, he has not received any reimbursement for this study. The other authors declare no conflicts of interest.

## Supporting information


Data S1.

## Data Availability

The data that support the findings of this study are available on request from the corresponding author. The data are not publicly available due to privacy or ethical restrictions.
