# Supplementary material for: High Injury Incidence Among Youth in the World's Largest Football Tournament
Source: Scand J Med Sci Sports. 2025 May 16;35(5):e70072. doi: 10.1111/sms.70072 (PMC12083556; doi:10.1111/sms.70072)
Supplement: Supplementary file 1 — Data S1. [file SMS-35-e70072-s001.docx]

**Supporting Information**

The tables show injury incidence rate ratio (IRRs) between age groups (S1), day of the tournament (S2) and sex (S3). The IRRs were calculated, according to Knowles et al.^[[1]](#endnote-1)^, as the ratio of two incidence rates (injuries/1000 player hours).


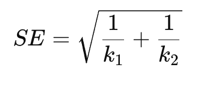


The standard error (SE) of the log (IRR) was estimated using the formula: , where k_1_ and k_2_ represent the number of injuries in the respective groups. The 95% confidence interval (CI) for the IRRs were calculated using the exponential of the log(IRR)±1.96xSE: Estimated 95% CI = exp (ln IRR ± 1.96 x SE (ln IRR)).

If the 95% CI did **not** include 1.00, the difference in injury rates was considered statistically significant. **Bold 95% CI** indicate a statistically significant difference between the groups.

**Table S1. IRRs with 95% CI between all age groups**

| **Age group** | **IRR** | **95% CI** |
| --- | --- | --- |
| 12 vs 11 | 0.93 | 0.70 – 1.24 |
| 13 vs 11 | 0.53 | **0.39 – 0.70** |
| 14 vs 11 | 0.59 | **0.45 – 0.76** |
| 15 vs 11 | 0.54 | **0.42 – 0.69** |
| 16 vs 11 | 0.51 | **0.39 – 0.66** |
| 17 vs 11 | 0.45 | **0.34 – 0.59** |
| 18 vs 11 | 0.42 | **0.31 – 0.59** |
| 13 vs 12 | 0.57 | **0.43 – 0.74** |
| 14 vs 12 | 0.63 | **0.50 – 0.79** |
| 15 vs 12 | 0.58 | **0.46 – 0.72** |
| 16 vs 12 | 0.55 | **0.43 – 0.69** |
| 17 vs 12 | 0.48 | **0.37 – 0.62** |
| 18 vs 12 | 0.46 | **0.34 – 0.62** |
| 14 vs 13 | 1.12 | 0.89 – 1.41 |
| 15 vs 13 | 1.02 | 0.82 – 1.28 |
| 16 vs 13 | 0.97 | 0.77 – 1.22 |
| 17 vs 13 | 0.85 | 0.66 – 1.10 |
| 18 vs 13 | 0.81 | 0.59 – 1.09 |
| 15 vs 14 | 0.91 | 0.76 – 1.09 |
| 16 vs 14 | 0.87 | 0.71 – 1.05 |
| 17 vs 14 | 0.76 | **0.61 – 0.95** |
| 18 vs 14 | 0.72 | **0.55 – 0.95** |
| 16 vs 15 | 0.95 | 0.79 – 1.14 |
| 17 vs 15 | 0.84 | 0.68 – 1.03 |
| 18 vs 15 | 0.79 | 0.60 – 1.03 |
| 17 vs 16 | 0.88 | 0.71 – 1.10 |
| 18 vs 16 | 0.83 | 0.63 – 1.10 |
| 18 vs 17 | 0.95 | 0.70 – 1.27 |

**Table S2. IRRs with 95% CI between all days in the tournament**

| **Day in the tournament** | **IRR** | **95% CI** |
| --- | --- | --- |
| 2 vs 1 | 1.66 | **1.34 – 2.05** |
| 3 vs 1 | 1.49 | **1.20 – 1.84** |
| 4 vs 1 | 1.78 | **1.45 – 2.18** |
| 5 vs 1 | 3.52 | **2.85 – 4.36** |
| 6 vs 1 | 4.26 | **2.96 – 6.14** |
| 3 vs 2 | 0.90 | 0.75 – 1.08 |
| 4 vs 2 | 1.07 | 0.90 – 1.27 |
| 5 vs 2 | 2.13 | **1.77 – 2.55** |
| 6 vs 2 | 2.57 | **1.82 – 3.64** |
| 4 vs 3 | 1.19 | **1.01 – 1.41** |
| 5 vs 3 | 2.36 | **1.97 – 2.83** |
| 6 vs 3 | 2.86 | **2.02 – 4.05** |
| 5 vs 4 | 1.98 | **1.68 – 2.35** |
| 6 vs 4 | 2.40 | **1.71 – 3.37** |
| 6 vs 5 | 1.21 | 0.86 – 1.71 |

**Table S3. IRR with 95% CI between sex**

| **Sex** | **IRR** | **95% CI** |
| --- | --- | --- |
| Boys vs girl | 0.88 | 0.78 – 1.00 |

1. Knowles SB, Marshall SW, Guskiewicz KM. Issues in estimating risks and rates in sports injury research. *J Athl Train.* 2006;41(2):207-215 [↑](#endnote-ref-1)
